# TRV–GFP: a modified *Tobacco rattle virus* vector for efficient and visualizable analysis of gene function

**DOI:** 10.1093/jxb/ert381

**Published:** 2013-11-11

**Authors:** Ji Tian, Haixia Pei, Shuai Zhang, Jiwei Chen, Wen Chen, Ruoyun Yang, Yonglu Meng, Jie You, Junping Gao, Nan Ma

**Affiliations:** Department of Ornamental Horticulture, China Agricultural University, Beijing 100193, China

**Keywords:** *Arabidopsis thaliana*, gene silencing, *Nicotiana benthamiana*, *Rosa* sp., TRV–GFP, VIGS.

## Abstract

Virus-induced gene silencing (VIGS) is a useful tool for functional characterization of genes in plants. Unfortunately, the efficiency of infection by *Tobacco rattle virus* (TRV) is relatively low for some non-Solanaceae plants, which are economically important, such as rose (*Rosa* sp.). Here, to generate an easy traceable TRV vector, a green fluorescent protein (GFP) gene was tagged to the 3’ terminus of the coat protein gene in the original TRV2 vector, and the silencing efficiency of the modified TRV–GFP vector was tested in several plants, including *Nicotiana benthamiana*, *Arabidopsis thaliana*, rose, strawberry (*Fragaria ananassa*), and chrysanthemum (*Dendranthema grandiflorum*). The results showed that the efficiency of infection by TRV–GFP was equal to that of the original TRV vector in each tested plant. Spread of the modified TRV virus was easy to monitor by using fluorescent microscopy and a hand-held UV lamp. When TRV–GFP was used to silence the endogenous *phytoene desaturas*e (*PDS*) gene in rose cuttings and seedlings, the typical photobleached phenotype was observed in 75–80% plants which were identified as GFP positive by UV lamp. In addition, the abundance of GFP protein, which represented the concentration of TRV virus, was proved to correlate negatively with the level of the *PDS* gene, suggesting that GFP could be used as an indicator of the degree of silencing of a target gene. Taken together, this work provides a visualizable and efficient tool to predict positive gene silencing plants, which is valuable for research into gene function in plants, especially for non-Solanaceae plants.

## Abstract (precis)

We developed an easy-traceable TRV vector, TRV2-GFP, by tagging a GFP to the coat protein. TRV2-GFP-infected plants could be identified efficiently by GFP monitoring. TRV2-GFP is useful for functional genomics in many plants, especially for non-*Solanaceae* plants, like rose

## Introduction

Virus-induced gene silencing (VIGS) is an attractive genetic tool for functional genomics in plants. Usually, VIGS targets a specific endogenous gene for silencing by constructing a recombinant virus carrying a fragment of the target gene (Kumagai *et al.*, 1995). Once the virus invades the host plant, the host defence machinery, such as post-transcriptional gene silencing (PTGS), will be activated and combat the virus. PTGS will target double-stranded RNA (dsRNA), which is commonly used for replication of most plant viruses, and lead to cleavage of dsRNA and sequential generation of small interfering RNA (siRNA). The siRNAs are loaded into the RNAi silencing complex (RISC) to degrade any endogenous transcript which shows sequence homology to the siRNA (Ruiz *et al.*, 1998; Baulcombe, 2004; Burch-Smith *et al*., 2004). Since VIGS is a transient method, it is an easy and rapid way to study the function of genes, even for those genes whose mutation is lethal. Moreover, despite stable plant transformation not being required for VIGS, VIGS was found to be able to be transmitted to progeny plants, namely non-integration-based transmissible PTGS (Senthil-Kumar and Mysore, 2011).

To date, VIGS vectors have been developed from several plant viruses, such as *Apple latent spherical virus* (ALSV) (Yamagishi and Yoshikawa, 2009; Sasaki *et al.*, 2011), *Barley stripe mosaic virus* (BSMV) (Bruun-Rasmussen *et al.*, 2007), and *Tobacco rattle virus* (TRV) (Liu *et al.*, 2002*b*). TRV is one of the most widely used vectors and has been successfully used in various plants, such as *Nicotiana benthamiana* leaf (Ratcliff *et al.*, 2001; Liu *et al.*, 2002*b*, 2004), *Arabidopsis* (Turnage *et al.*, 2002), tomato fruit (Liu *et al.*, 2002*a*; Fu *et al.*, 2005), pepper leaf (Chung *et al.*, 2004), petunia flower (Chen *et al.*, 2004), *Aquilegia* (Gould and Kramer, 2007), *Thalictrum* flower (Di Stilio *et al.*, 2010), rose flower (Ma *et al.*, 2008), strawberry fruit (Jia *et al.*, 2011), California poppy (Wege *et al.*, 2007), *Jatropha curcas* (Ye *et al.*, 2009), and *Mirabilis jalapa* (Singh *et al.*, 2012).

Since VIGS vectors were developed from plant viruses, application of VIGS is species dependent. For instance, the TRV vector is able to silence genes in all tested Solanaceae plants, such as *N. benthamiana*, tomato, pepper, and petunia. It is, however, less effective in some non-Solanaceae plants crops, such as rose (Ma *et al.*, 2008), which is an important economic and cultural ornamental crop throughout the world.

In the present work, a modified TRV vector, TRV–GFP, was generated by fusing the enhanced green fluorescent protein (EGFP) open reading frame (ORF) sequence to the 3’ terminus of the coat protein (CP) gene in the TRV2 plasmid. The TRV–GFP vector was tested in several plants, including *N. benthamiana*, *Arabidopsis*, rose, strawberry, and chrysanthemum. The results showed that TRV–GFP could be detected by using a hand-held UV lamp, and the efficiency of infection by TRV–GFP was equal to that of the original TRV vector. This work provides an effective tool for monitoring the efficiency of VIGS in functional genomics of non-Solanaceae plants.

## Materials and methods

### Plant materials

*Nicotiana benthamiana* and wild-type *Arabidopsis* ecotype Col-0 plants were grown in pots in a growth chamber at 23 °C with a 16h/8h photoperiod and ~60% relative humidity.

For cuttings, *Rosa* sp. cv. ‘Samantha’ branches were rooted for 4 weeks in vermiculite under intermittent mist. After vacuum infiltration, the plants were transplanted into plastic pots containing a steam-sterilized medium consisting of peat:horticultural grade perlite:washed silica (1:1:1) and were grown in a growth chamber (23 °C, 16/8h photoperiod, ~60% relative humidity).

For rose seedlings, seeds of *Rosa* sp. cv. ‘Magic Meidiland’ were stored in wet sand at 1 °C for 2 months, and then were transferred to plates to geminate at room temperature. The VIGS experiment was conducted when the seedlings had two cotyledons, then they were transplanted into pots containing a mixture of 1:1 (v/v) peat:vermiculite, and transferred to a greenhouse (23°C, relative humidity 60–70%, 16h/8h light/dark).

For strawberry, *Fragaria×ananassa* cv. ‘Benihoppe’ plants were used. The plants were propagated by *in vitro* culture for 65 d. After vacuum infiltration treatment, strawberry plants were rinsed with distilled water and then transplanted into pots containing a mixture of 1:1 (v/v) peat:vermiculite. The plants were grown in a greenhouse (23 °C, relative humidity 60–70%, 14h/10h light/dark cycles).

For chrysanthemum (*Dendranthema grandiflorum*), a popular ground cover-type cultivar ‘Fall color’ (hereafter, chrysanthemum), was used. Plants were propagated by *in vitro* culture for 2 months, then transplanted into pots containing a mixture of 1:1 (v/v) peat:vermiculite, and transferred to a greenhouse (23 °C, relative humidity 60%–70%, 16h/8h light/dark).

### Vector construction

For generation of TRV–GFP, the *EGFP*-tagged *CP* fragment was generated by overlap PCR and was inserted into the pTRV2 vector digested by *Hin*dIII and *Bam*HI.

For silencing the *phytoene desaturas*e (*PDS*) gene in *N. benthamiana*, a 368bp fragment of the *N. benthamiana NtPDS* gene (EU165355) was amplified and cloned into pTRV2/pTRV2-GFP to form pTRV2-*NtPDS*/pTRV2-GFP-*NtPDS*. For silencing *PDS* in *A. thaliana*, a 1000bp fragment of the *Arabidopsis AtPDS* gene (NM117498) was amplified and was cloned into pTRV2/pTRV2-GFP to form pTRV2-*AtPDS*/pTRV2-GFP-*AtPDS*. For silencing *PDS* in rose, a 486bp fragment of the *RhPDS* (RU05877, Rose Transcriptome Database, http://bioinfo.bti.cornell.edu/cgi-bin/rose_454/index.cgi) gene was amplified from rose cDNA and cloned into pTRV2/pTRV2-GFP to form pTRV2-*RhPDS*/pTRV2-GFP-*RhPDS*. The primers used are listed in Supplementary Table S1 available at *JXB* online.

### Agroinfiltration and vacuum infiltration

The *Agrobacterium* strain GV3101 containing TRV-VIGS vectors was grown at 28 °C in Luria–Bertani medium supplemented with 10mM MES, 20mM acetosyringone, 50mg l^–1^ gentamicin, and 50mg l^–1^ kanamycin for ~24h. *Agrobacterium* cells were harvested and suspended in the infiltration buffer (10mM MgCl_2_, 200mM acetosyringone, and 10mM MES, pH 5.6). A mixture of *Agrobacterium* cultures containing pTRV1 and pTRV2 and its derivatives, in a ratio of 1:1 (v/v), were placed in the dark at room temperature for 4h before inoculation. For vacuum infiltration, Silwet L-77 was added to a final concentration of 0.01% (v/v).

*Nicotiana benthamiana* infiltration was performed as described by Liu *et al.* (2002*a*). *Agrobacterium* cultures containing pTRV1 and pTRV2 or its derivatives (1:1, OD_600_=1.0) were injected into the lower leaf of four-leaf stage plants by using a 1ml needleless syringe. For axil injection, 20 μl of bacterial suspension (1:1, OD_600_=4.0) were injected per plant into the leaf axil using a 1ml syringe with a needle at the four-leaf stage period.

Since rose leaves and axils are hard to inject, the vacuum infiltration method was used for rose cuttings, and seedlings were infiltrated as described previously (Ma *et al.*, 2008; Yan *et al.*, 2012). Four-week-old rose cuttings were removed from the soil, the roots were rinsed with distilled water, and whole plants were submerged in infiltration mixture containing pTRV1 and pTRV2 or its derivatives (OD_600_=1.0) and subjected to a vacuum at –25 kPa twice, each for 60 s. This was repeated once. Treated plants were briefly washed with distilled water and then planted in pots. For seedlings, the seeds generated were submerged in infiltration mixture containing pTRV1 and pTRV2 or its derivatives (OD_600_=1.0) and subjected to a vacuum at –25 kPa twice, each for 60 s. The infiltrated seeds were briefly washed with distilled water and placed on wet filter paper for several days, and then the seedlings were planted in pots.

Strawberry was vacuum infiltrated in the same way as rose cuttings and seedlings. After the release of the vacuum, the plants were briefly washed with distilled and planted in pots.

*Arabidopsis* and chrysanthemum infiltration was performed as described by Burch-Smith *et al.* (2006). A mixture of *Agrobacterium* culture containing pTRV1 and pTRV2-GFP (OD_600_=1.5) was infiltrated into the two leaves of 2-week-old *Arabidopsis* plants and the lower leaves of six- to eight-leaf-stage chrysanthemum plants using a needleless syringe. The infected chrysanthemum plants were transferred into pots.

### Total RNA extraction and RT–PCR

Total RNA was extracted from control and infected plants individually and treated with RNase-free DNase I (Promega, USA). First-strand cDNA was synthesized using 1 μg of total RNA with TRV-specific primers (pYL156 R for TRV1 and OYL198 for TRV2), and oligod(T) (for target genes) or random primer (for *CP-GFP*). Semi-quantitative reverse transcription–PCR (RT–PCR) was replicated for at least three biological replicates. The PCR products were separated on a 1.2% agarose gel and the images were scanned and analysed using AlphaImager 2200 (Alpha Innotech, San Leandro, CA, USA). The primers used are listed in Supplementary Table S1 at *JXB* online.

### GFP imaging

GFP imaging in *N. benthamiana* cells was conducted using a laser scanning confocal microscope (Nikon C1). For visualization of the whole plant, the samples were illuminated with a 100W hand-held long-wave ultraviolet lamp (UV products, Upland, CA, USA; Black Ray model B 100AP/R) and were photographed using a Kodak wratten filter 15.

### Western blot

Western blot analysis was performed using GFP-specific antibody (Abcam Inc.). Protein was extracted from leaves of individual plant of *N. benthamiana*, *A. thaliana*, rose, and strawberry with 300 μl of extraction buffer [100mM TRIS, pH 6.8, 2.5% SDS, 100mM dithiothreitol (DTT), 100mM NaCl, 10% glycerol]. For *Arabidopsis*, protein was extracted from leaves of individual plants with 300 μl of extraction buffer (125mM TRIS-HCl, pH 6.8, 4% SDS, 20% glycerol, 2% β-mercaptoethanol). For chrysanthemum, protein was extracted from leaves of individual plants with 300 μl of extraction buffer [200mM HEPES, pH 8.0, 20mM EDTA, 100mM DTT, 200mM phenylmethylsulphonyl fluoride (PMSF), 0.2mg ml^–1^ leupeptin, 50mg ml^–1^ Polyclar VT]. Bradford assays were used to determine protein quantities, and equal amounts of proteins (10 μg) were separated by 10% SDS–PAGE. Proteins were transferred to a polyvinylidene difluoride membrane (GE Healthcare). CP–GFP was detected after an overnight incubation at room temperature with a 1:10 000 dilution of an anti-GFP antibody conjugated to alkaline phosphatase (Bedoya *et al*., 2010). Alkaline phosphatase was detected using a chemiluminescent substrate (CSPD; Roche) and exposed to X-ray film (Kodak X-OMAT BT Film/XBT-1).

## Results

### Validation of the TRV–GFP vector in *Nicotiana benthamiana*

**Fig. 1.**
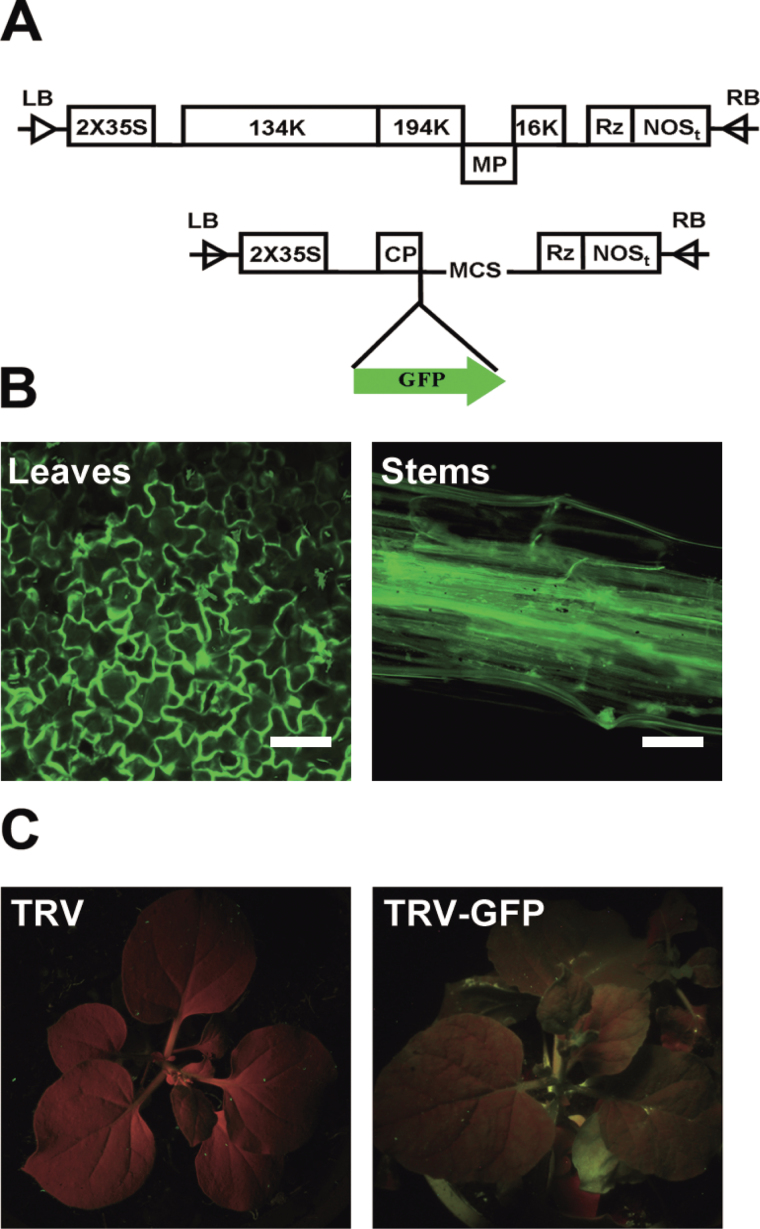
Construction and validation of the TRV–GFP vector. (A) Schematic diagram of the TRV–GFP vector. (B) GFP imaging of TRV–GFP-infiltrated *N. benthamiana* by fluorescence microscopy. (C) Imaging of TRV- (left) and TRV–GFP- (right) infiltrated *N. benthamiana* by a UV lamp. The TRV 5′- and 3′-untranslated regions (UTRs) are indicated by lines. Open boxes, TRV 2×35S promoter, Rz (self-cleaving ribozyme), nopaline synthase terminator (NOSt), and CP. Green arrow, GFP cistron,. Scale bar=100mm.

In order to generate a visualizable TRV vector, the stop codon of the ORF of CP in the TRV2 vector was deleted and the vector was tagged with the EGFP ORF sequence by overlap PCR. The modified version of the TRV2 vector was named TRV–GFP (Fig. 1A). The empty TRV–GFP was transferred into *Agrobacterium* strain 3101 and introduced into *N. benthamiana* plants by injection on leaves using a needleless syringe. As illustrated in Fig. 1B, GFP fluorescence was observed in injected leaves including stems at 5 dpi (days post-inoculation), indicating that the virus spread in the plants. At 7 dpi, GFP fluorescence could be observed in the upper young leaves using a long-wave UV lamp (Fig. 1C).

**Fig. 2.**
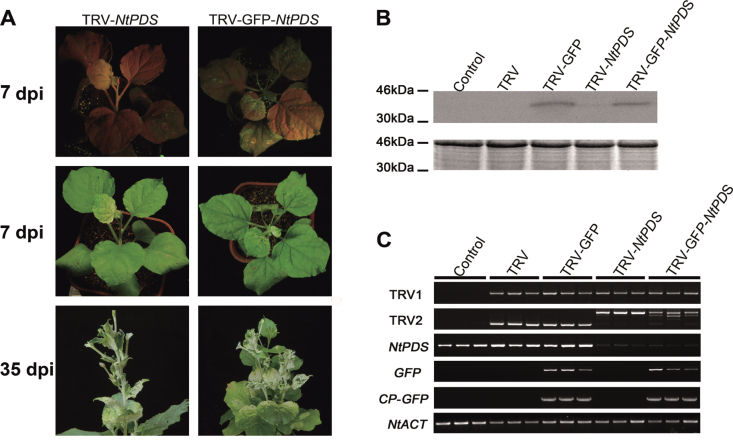
Validation of the TRV–GFP vector in *N. benthamiana*. (A) TRV- and TRV–GFP-infiltrated *N. benthamiana* plants. The four-leaf-stage plants were inoculated on a leaf with a mixture of pTRV2-*PDS*/pTRV2-GFP-*PDS* and pTRV1 (1:1, OD_600_=1.0) using a needleless syringe. Plants were photographed under UV illumination and normal light. (B) CP–GFP protein levels in upper leaves of *N. benthamiana* 7 d after TRV vector inoculation. A 10 μg aliquot of protein was used for western blot in each lane, and anti-GFP was used as antibody to detect CP–GFP fusion protein. Coomassie blue staining was used for confirmation of equal loading in each lane. (C) Semi-quantitative RT–PCR of *TRV1, TRV2*, *NtPDS*, *GFP, a*nd *CP*-*GFP* in control and inoculated *N. benthamiana* plants. *NtACT* was used as an internal control. The TRV2 fragment was larger in plants infected by TRV-*NtPDS* and TRV–GFP-*NtPDS* due to the inserted *NtPDS* fragment.

The efficiency of TRV–GFP and TRV vectors in silencing the endogenous *PDS* in *N*. *benthamiana* was compared. As expected, GFP fluorescence could be observed in the upper young leaves by using a long-wave UV lamp at 7 dpi (Fig. 2A). *Nicotiana benthamiana* plants infected by TRV-*NtPDS* exhibited the typical photobleached phenotype in the top young leaves at 7 dpi with a silencing efficiency of 97.9% (Table 1). The phenotype developed and lasted until flowering (35 dpi) (Fig. 2A). Plants infected by TRV–GFP-*NtPDS* also exhibited a very similar phenotype to that of plants infected by TRV-*NtPDS.* Furthermore, the percentage of positive plants for TRV–GFP-*NtPDS* (95.8%) was very close to that for TRV*-NtPDS* (97.9%) (Table 1), indicating that GFP tagging did not alter the gene silencing ability of the TRV vector.

**Table 1.** Silencing efficiency of TRV and TRV–GFP in N. benthamiana

	TRV	TRV–GFP
Total	48	48
GFP positive	–	46
PDS silencing	47	46
GSEI	97.9%	95.8%
GSEG	–	100.0%

GSEI, gene silencing efficiency of all infected plants; GSEG, gene silencing efficiency of GFP-positive plants.

Using western blot, GFP protein was detected in the top young leaves in TRV–GFP- and TRV–GFP-*NtPDS*-infected plants, but was not seen in TRV- or TRV-*NtPDS*-infected plants, further supporting the movement of the modified TRV–GFP virus (Fig. 2B). Semi-quantitative RT–PCR showed that the abundance of endogenous *NtPDS* transcript was greatly decreased in both TRV–GFP-*NtPDS*- and TRV*-NtPDS*- infected plants, indicating that the modified TRV–GFP vector was as effective as the original TRV (Fig. 2C). In addition, *N. benthamiana* plants were infected by TRV–GFP using the axil injection method (for details, see the Materials and methods). Similarly, the effects of the TRV–GFP vector were indistinguishable from those of the original TRV vector (Supplementary Fig. S1 at *JXB* online). Collectively, the modification of TRV–GFP does not jeopardize the function of TRV in gene silencing.

**Fig. 3.**
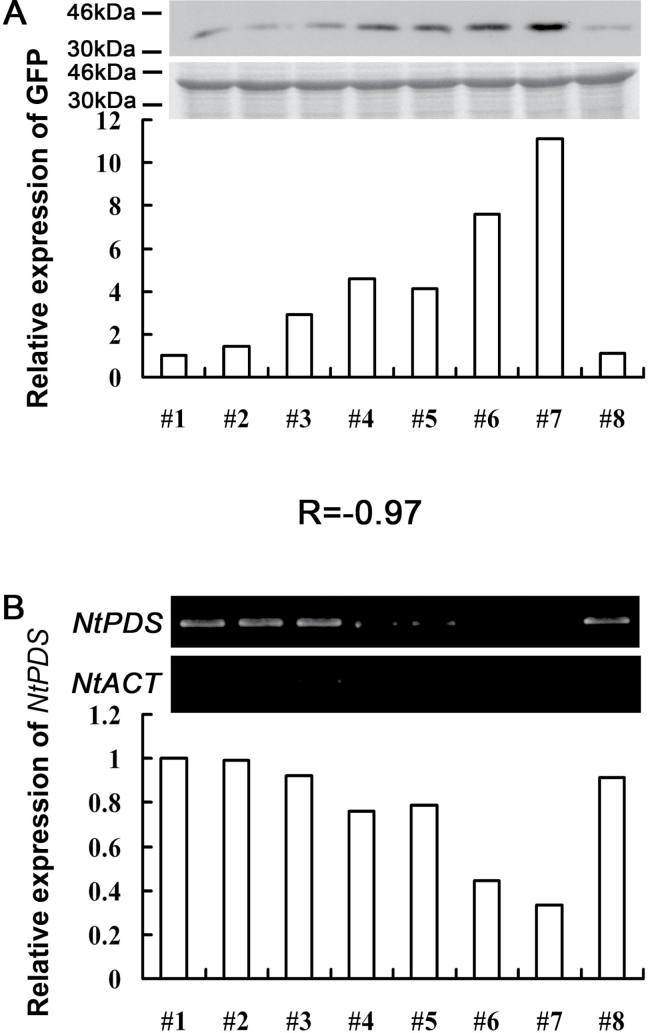
Correlation between CP–GFP protein level and abundance of the *NtPDS* gene in TRV–GFP-*NtPDS*-infected *N. benthamiana* plants. (A) Level of accumulation of CP–GFP protein. Protein was extracted from the third leaf above the inoculated leaves at 7 dpi. A 10 μg aliquot of protein was used for western blot in each lane and anti-GFP was used to detect CP–GFP fusion protein. Molecular sizes are indicated. Coomassie blue staining was used for confirmation of equal loading in each lane. (B) Abundance of the *NtPDS* gene. Total RNA was extracted from the top young leaves of the *N. benthamiana* plant which was used for western blot analysis at 14 dpi. *NtACT* was used as an internal control. The expression level of CP–GFP protein and the *NtPDS* gene was quantified by AlphaImager 2200. *R* is the correlation coefficient. Numbers indicate individual plants. The CP–GFP protein and *NtPDS* expression level in plant #1 was defined as 1, respectively.

Since GFP protein was fused to the CP protein in the TRV–GFP vector, it was assumed that the accumulation level of GFP protein might reflect the concentration of the TRV virus in plants and indicate the degree of gene silencing, because a higher concentration of virus usually corresponds to the degree of gene silencing. As shown in Fig. 3, the relationship between the level of GFP protein and gene silencing efficiency was analysed, and it was found that the concentration of CP–GFP protein, which indicated the virus concentration, was negatively related to the abundance of the *NtPDS* gene [correlation coefficient (*r*)= –0.97].

Collectively, these results indicted that GFP protein fused with CP protein does not affect the silencing efficiency of the modified TRV–GFP vector, although the movement of the TRV–GFP virus is somewhat slower than that of the original TRV vector. Furthermore, the level of CP–GFP protein could be used to evaluate the degree of silencing of the target gene, which would further increase the efficiency of the VIGS approach.

### Validation of the TRV–GFP vector in *Arabidopsis thaliana*

The TRV vector has been successfully used for functional genomics of *A. thaliana* (Burch-Smith *et al.*, 2006). To investigate whether the TRV–GFP vector could induce gene silencing in *A. thaliana* plants, an attempt was made to suppress the expression of the endogenous *PDS* gene in *A. thaliana*. A mixture of *Agrobacterium* cultures containing pTRV2-*AtPDS*/pTRV2-GFP-*AtPDS* and pTRV1 was infiltrated onto the entire leaf of two- to three-leaf-stage plants.

**Fig. 4.**
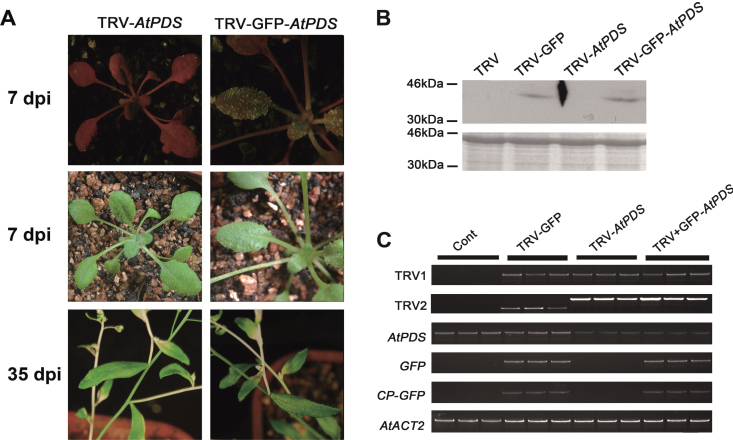
Validation of the TRV–GFP vector in *A. thaliana*. (A) TRV- and TRV–GFP-infiltrated plants. Plants were photographed under UV illumination and normal light. (B) CP–GFP protein levels in upper leaves 7 d after TRV vector inoculation. A 10 μg aliquot of protein was used for western blot in each lane and anti-GFP was used as antibody to detect CP–GFP fusion protein. Coomassie blue staining was used for confirmation of equal loading in each lane. (C) Semi-quantitative RT–PCR of *TRV1, TRV2*, *AtPDS*, *GFP*, and *CP*-*GFP* in control and inoculated *A. thaliana* plants. *AtACT* was used as an internal control. The TRV2 fragment was larger in plants infected by TRV-*AtPDS* and TRV–GFP-*AtPDS* due to the inserted *AtPDS* fragment.

**Fig. 5.**
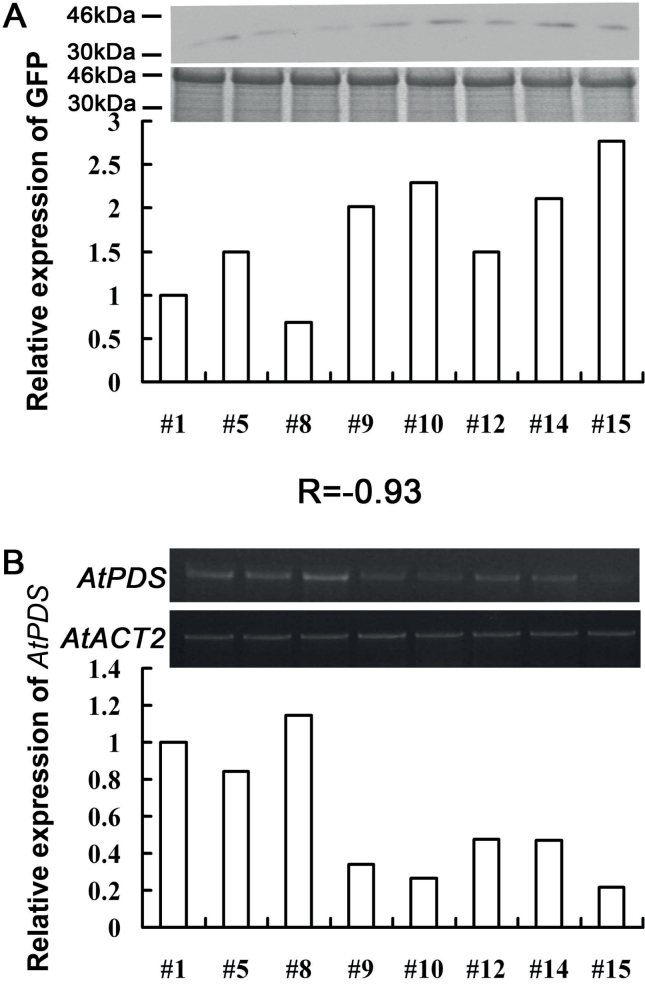
Correlation between CP–GFP protein level and abundance of the *AtPDS* gene in TRV–GFP-*AtPDS*-infected *A. thaliana* plants. (A) Level of accumulation of CP–GFP protein. Protein was extracted from the third leaf above the inoculated leaf at 7 dpi. A 10 μg aliquot of protein was used for western blot in each lane and anti-GFP was used to detect CP–GFP fusion protein. Molecular sizes are indicated. Coomassie blue staining was used for confirmation of equal loading in each lane. (B) Abundance of the *AtPDS* gene. Total RNA was extracted from the top young leaves of the *A. thaliana* plant which was used for western blot analysis at 14 dpi. *AtACT* was used as an internal control. The expression level of CP–GFP protein and the *AtPDS* gene was quantified by AlphaImager 2200. *R* is the correlation coefficient. Numbers indicate individual plants. The CP–GFP protein and *AtPDS* expression level in plant #1 was defined as 1, respectively.

About 7 d after agroinfiltration, GFP fluorescence was observed in the upper young leaves and injected leaves (Fig. 4A). A typical photobleached phenotype was observed in the inflorescence stem of *Arabidopsis* plants infected with pTRV2-GFP-*AtPDS* and pTRV2-*AtPDS* at 30 d after infiltration (Fig. 4A). Western analysis confirmed that GFP proteins were only accumulated in TRV–GFP- and TRV–GFP-*AtPDS*-infected plants by using GFP antibody (Fig. 4B). Consistently, semi-quantitative RT–PCR showed that the *AtPDS* level was substantially reduced in both TRV-*AtPDS*-and TRV–GFP-*AtPDS*-infiltrated plants when compared with plants infiltrated with TRV and TRV–GFP (Fig. 4C). As in *N. benthamiana*, it was found that the GFP protein accumulation was also negatively correlated with the expression level of the target gene in *A. thaliana* [correlation coefficient (*r*)= –0.93] (Fig. 5). In addition, the silencing efficiency of pTRV2-GFP-*AtPDS* (93.8% photobleached phenotype) was very close to that of pTRV2-*AtPDS* (95.8%), indicating that the modified TRV vector was not different from the original vector (Table 2).

**Table 2.** Infection efficiency of TRV and TRV–GFP in A. thaliana

	TRV	TRV–GFP
Total	48	48
GFP positive	–	45
PDS silencing	46	45
GSEI	95.83%	93.75%
GSEG	–	100.00%

GSEI, gene silencing efficiency of all infected plants; GSEG, gene silencing efficiency of GFP-positive plants.

### Application of the TRV–GFP vector in rose (*Rosa* sp.)

Rose (*Rosa* sp.) is an important horticultural crop both economically and culturally. Unfortunately, the infection efficiency of TRV is relatively low in rose, which means that it is more time-consuming to identify positive TRV-infected plants. Since TRV–GFP infection was visualizable, the use of TRV–GFP in rose was tested.

**Fig. 6.**
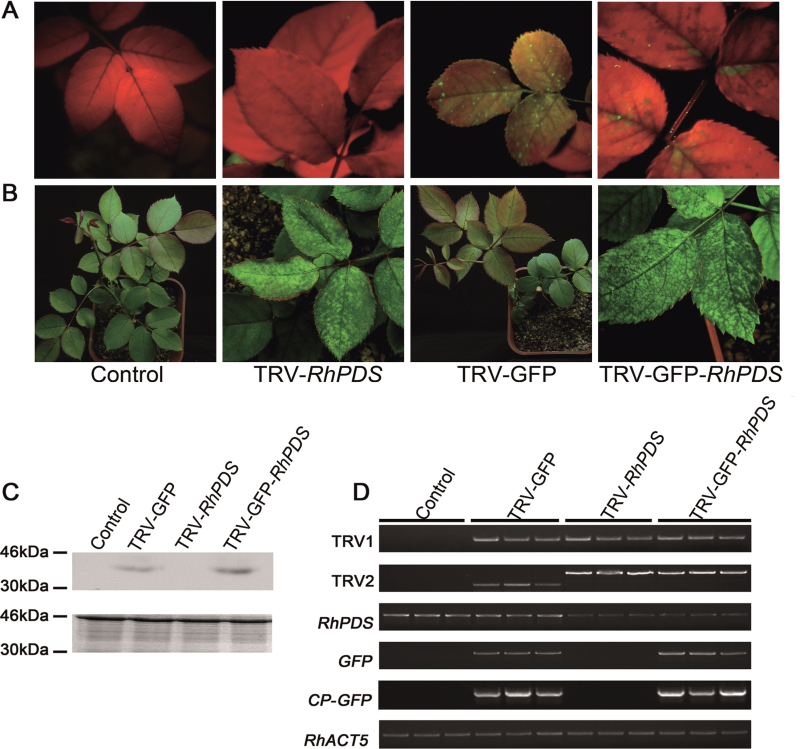
Silencing of *RhPDS* in rose cuttings. (A) TRV- and TRV–GFP-infiltrated rose plants. Rose cuttings were inoculated with a mixture of pTRV2-*RhPDS*/pTRV2-GFP-*RhPDS* and pTRV1 (1:1, OD_600_=1.0) using vacuum infiltration. The plants were photographed under UV illumination and normal light at 30 and 45 dpi, respectively. (B) CP–GFP protein levels in the upper leaves 30 d after inoculation. A 10 μg aliquot of protein was used for western blot in each lane and anti-GFP was used to detect CP–GFP fusion protein. Coomassie blue staining was used for confirmation of equal loading in each lane. (C) Semi-quantitative RT–PCR of *TRV1*, *TRV2*, *RhPDS*, *GFP*, and *CP-GFP* in control and inoculated plants. *RhACT5* was used as an internal control. Total RNA and protein were extracted from the top young leaves at 45 dpi, which did not exist when the cuttings were infiltrated and grew out at ~15 dpi.

At ~30 dpi, green fluorescence was observed in the top young leaves of pTRV2-GFP-*RhPDS*-infected rose plants (Fig. 6A). The typical photobleached phenotype appeared in the top young leaves at ~45 dpi, which was slower than that in *N. benthamiana* (Fig. 6B). Semi-quantitative RT–PCR and western blot showed the existence of the *CP-GFP* transcript and CP–GFP fusion protein in the top young leaves of plants infected by TRV–GFP and TRV–GFP-*RhPDS*, indicating that the modified TRV virus successfully moved to the newly emerged leaves (Fig. 6C, D). Also, the abundance of the *RhPDS* transcript was substantially reduced in the top young leaves of plants infected by TRV–GFP and TRV–GFP-*RhPDS* (Fig. 6D). Moreover, detached rose petals were infected using the vacuum infiltration method, and the results demonstrated that GFP fluorescence was very strong in petals at 7 dpi (Supplementary Fig. S2 at *JXB* online). Since rose petals are important for investigating floral scent and colouring, TRV–GFP would be valuable for characterization of genes which are involved in these biological processes.

**Fig. 7.**
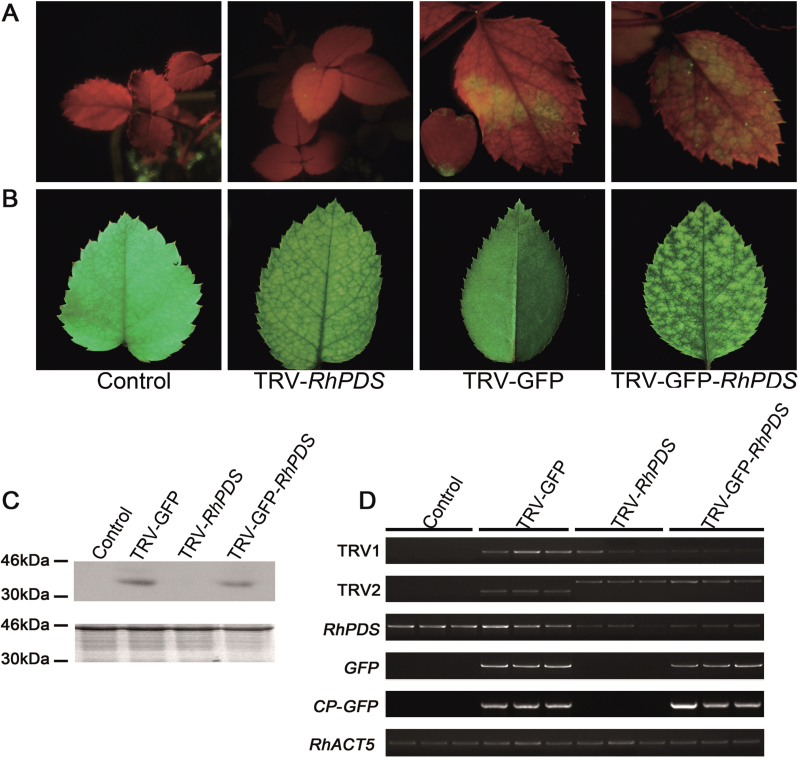
Silencing of *RhPDS* in rose seedlings. (A) TRV- and TRV–GFP-infiltrated rose plants. Rose seedlings were inoculated with a mixture of pTRV2-*RhPDS*/pTRV2-GFP-*RhPDS* and pTRV1 (1:1, OD_600_=1.0) using vacuum infiltration. The plants were photographed under UV illumination and normal light at 25 and 30 dpi, respectively. The inset image in the fluorescent photo of TRV–GFP-infected plant shows a petal with GFP green fluorescence. (B) CP–GFP protein levels in upper leaves 30 d after TRV vector inoculation. A 10 μg aliquot of protein was used for western blot in each lane and anti-GFP was used as antibody to detect CP–GFP fusion protein. Coomassie blue staining was used for confirmation of equal loading in each lane. (C) Semi-quantitative RT–PCR of *TRV1*, *TRV2*, *RhPDS*, *GFP*, and *CP-GFP* in control and inoculated plants. *RhACT5* was used as an internal control. Total RNA and protein were extracted from the top young leaves.

A previous report showed that the VIGS approach could be used to silence target genes in seeds to investigate the gene function in the earlier development period (Yamagishi and Yoshikawa, 2009; Senthil-Kumar and Mysore, 2011). Thus experiments were carried out to test whether the modified TRV–GFP was also compatible with rose seeds. After 2 months storage at 1 °C in wet sand, seeds of *Rosa* sp. cv. ‘Magic Meidiland’ were placed on wet paper in a plate to geminate at room temperature. Germinated seeds were used to perform the vacuum agroinfiltration. As shown in Fig. 7A, GFP fluorescence was observed in the top young leaves at ~25 dpi. Interestingly, when the plants were in bloom, GFP fluorescence could even be observed in petals (Fig. 7A, inset).

The existence of CP–GFP in the top leaves of rose seedlings was confirmed by semi-quantitative RT–PCR and western blot (Fig. 7 C, D). As expected, the photobleached phenotype was observed in the top young leaves at 30 dpi, and the abundance of *RhPDS* was substantially reduced in plants infected by TRV-*RhPDS* and TRV–GFP-*RhPDS* (Fig. 7D).

**Fig. 8.**
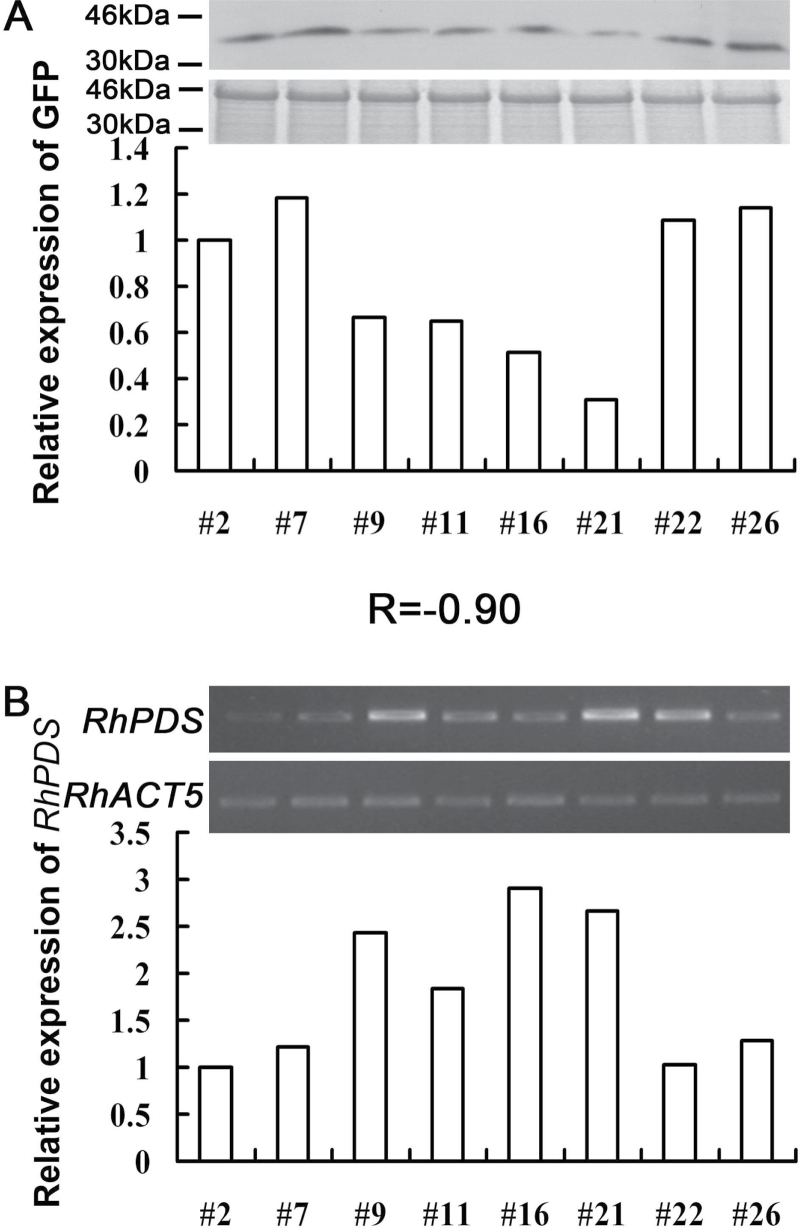
Correlation between CP–GFP protein level and abundance of the *RhPDS* gene in TRV–GFP-*RhPDS*-infected rose cuttings. (A) Accumulation level of CP–GFP protein. Protein was extracted from the top young leaves at 45 dpi. A 10 μg aliquot of protein was used for western blot in each lane and anti-GFP was used as antibody to detect CP–GFP fusion protein. Coomassie blue staining was used for confirmation of equal loading in each lane. (B) Abundance of the *RhPDS* gene. Total RNA was extracted from the top young leaves of the rose plant which was used for western blot analysis at 45 dpi. *RhACT5* was used as an internal control. The expression level of CP–GFP and *RhPDS* was quantified by AlphaImager 2200. *R* is the correlation coefficient. Numbers indicate individual plants. The expression level of CP–GFP protein and the *RhPDS* gene in plant #2 was defined as 1, respectively.

The number of plants positively infected by TRV and TRV–GFP was compared. A *PDS*-positive plant was defined as one that exhibited the typical photobleached phenotype and a substantially decreased abundance of *RhPDS* transcript, while GFP-positive plants were defined as plants exhibiting GFP fluorescence under UV light. The percentage of *PDS*-positive plants was similar between TRV-*RhPDS* (33.3% for cuttings and 34.3% for seedlings) and TRV–GFP-*RhPDS* (34% for cuttings and 29.4% for seedlings) plants, indicating that fusion with GFP did not obviously influence the silencing efficiency of the TRV vector in rose (Table 3). For GFP-positive plants, 80% (for cuttings) and 75% (for seedlings) of plants appeared to be *PDS* positive (Table 3). These results indicated that application of TRV–GFP could greatly increase the screening efficiency when compared with the original TRV vector, especially for plants which are less compatible with the TRV virus, such as rose. Interestingly, it was found that the photobleached phenotype appeared earlier in seedlings (30 dpi) than in cuttings (45 dpi). Moreover, as proved in *N. benthamiana* and *A. thaliana*, it was found that the level of CP–GFP protein was negatively correlated with the abundance of *RhPDS* transcript in the top leaves of rose cuttings infected by TRV–GFP-*RhPDS* [correlation coefficient (*r*)= –0.90] (Fig. 8).

**Table 3.** Silencing efficiency of TRV and TRV–GFP in Rosa sp

		TRV	TRV–GFP
Cuttings	Total	30	35
	GFP positive	–	15
	PDS silencing	9	12
	GSEI	33.3%	34.0%
	GSEG	–	80.0%
Seedlings	Total	32	51
	GFP positive	–	20
	PDS silencing	11	15
	GSEI	34.3%	29.4%
	GSEG	–	75.0%

GSEI, gene silencing efficiency of all infected plants; GSEG, gene silencing efficiency of GFP-positive plants.

These results further supported that it is possible to estimate th edegree of gene silencing by detecting the level of CP–GFP protein in the earlier period of VIGS. Considering that the photobleached phenotype appeared at ~30 dpi in rose cuttings, the modified TRV–GFP vector could be a valuable tool to increase the efficiency of reverse genetic research greatly in rose.

### Application of the TRV–GFP vector in other plants

**Fig. 9.**
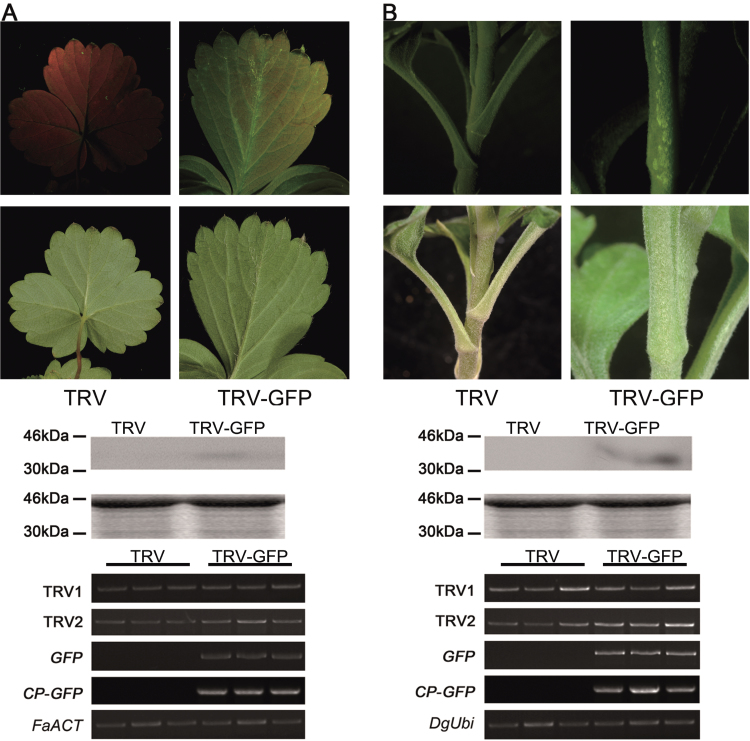
Application of TRV–GFP in strawberry (A) and chrysanthemum (B). The plants were inoculated by needleless vacuum infiltration (for strawberry) or syringe injection (for chrysanthemum). The plants were photographed at 21 dpi (for strawberry) and 14 dpi (for chrysanthemum). A 10 μg aliquot of protein was used for western blot in each lane and anti-GFP was used as antibody to detect CP–GFP fusion protein. Coomassie blue staining was used for confirmation of equal loading in each lane. The *FaACT* and *DgUbi* genes were used as internal controls for RT–PCR in strawberry and chrysanthemum, respectively.

To confirm whether the modified TRV–GFP is suitable for other plants, the TRV–GFP vector was tested in two important horticultural crops, strawberry and chrysanthemum. For strawberry, plants generated from tissue culture were infiltrated by the vacuum infiltration method (1:1, OD_600_=1.0). For chrysanthemum, the plants were inoculated using a needleless syringe (1:1, OD_600_=1.5). GFP fluorescence signals were strong in the top new leaves above the agroinoculated leaves at ~21 dpi in strawberry (Fig. 9A). For chrysanthemum, GFP fluorescence was stronger in stems at ~14 dpi (Fig. 9B). As expected, RT–PCR and western blot confirmed that TRV–GFP could successfully work in strawberry and chrysanthemum (Fig. 9; Table 4). Collectively, these results indicated that the TRV–GFP vector could be used widely in multiple plants.

**Table 4.** Infection efficiency of TRV and TRV–GFP in strawberry and chrysanthemum

Plant		TRV	TRV–GFP
Strawberry	Total	–	65
	GFP positive	–	24
	Infection efficiency	–	36.9%
Chrysanthemum	Total	–	24
	GFP positive	–	17
	Infection efficiency	–	70.8%

## Discussion

In the post-genomic era, more and more efforts have been made in functional genomics. As a simple and rapid tool in functional genomics, VIGS has been widely used to analyse gene function in many plant species, as well as in multiple development processes and in response to extrinsic stimuli. Therefore, in the past decade, tremendous improvements VIGS have been reported, such as developing new vectors and improving the vector delivery methods (Jiang *et al.*, 2011; Senthil-Kumar and Mysore, 2011).

Since VIGS vectors are all constructed based on natural plant viruses, application of certain VIGS vectors would be limited by species specificity. The TRV vector is the most widely used VIGS vector so far. It is applicable for many plants because the virus has a broad host range (Bachan and Dinesh-Kumar, 2012). However, the efficiency of TRV in some non-Solanaceae plants, such as rose and strawberry, was still relatively low when compared with *N. benthamiana*, petunia, and tomato (Ma *et al.*, 2008; personal communication).

Here, to increase the selection efficiency, the CP gene in TRV2 was tagged with GFP to generate a TRV–GFP vector. This modified vector exhibited similar efficiency in infection and gene silencing in *N. benthamiana* and *A. thaliana*, although the obvious phenotype of gene silencing emerged a little bit later in TRV–GFP-infected *N. benthamiana* plants than in plants infected with the original TRV vector. In rose, TRV–GFP was tested in cuttings and seedlings by vacuum infiltration methods. The results indicated that TRV–GFP could spread and silence endogenous genes as efficiently as the original TRV vector. Given that TRV–GFP could be readily tracked by a UV lamp, it is easy to identify GFP-positive plants from the TRV–GFP-infected population. Moreover, ~80% of GFP-positive plants were shown to be positive for gene silencing. Therefore, the modified TRV–GFP vector could promote screening efficiency, although it could not increase the authentic efficiency of gene silencing.

Previously, a dark purple transgenic tomato line was generated by overexpressing an anthocyanin regulatory gene from maize, *Lc* (*Leaf colour*) (Goldsbrough *et al.*, 1996). It was reported that this *Lc*-based tomato line was successfully used as a functional analysis tool to indicate the effects of VIGS by silencing the *Lc* gene (Jiang *et al.*, 2008). Similarily, a MYB-related transcription factor gene from *Antirrhinum majus*, *Rosea1* (*Ros1*), which is able to activate anthocyanin biosynthesis, was also introduced into tomato under the control of the fruit-specific *E8* promoter. The generated transgenic tomato line exhibited a specific phenotype of purple fruit. Then, a modified TRV2 vector carrying a fragment of *Ros1* was used to perform VIGS analysis in the transgenic tomato, and the disappearance of purple colour could be used as a marker of gene silencing (Orzaez *et al.*, 2009). *Ros1* was also tagged to several viruses, including *Tobacco etch potyvirus* (TEV), *Tobacco mosaic virus* (TMV), *Potato virus X* (PVX), and *Turnip mosaic virus* (TuMV), and served as a marker to track virus infection and movement (Bedoya *et al.*, 2012). Moreover, the concentration of anthocyanin was used to determine the viral load qualitatively and quantitatively. Interestingly, although *Ros1* was tagged to TRV and TRV-*Ros1* induced symptoms of virus infection, the symptomatic tissue failed to show any phenotype of anthocyanin accumulation (Bedoya *et al.*, 2012).

Although anthocyanin accumulation is readily detectable by the naked eye, it is an important trait for many plants, especially for ornamental plants, such as petunia, rose, and chrysanthemum. Therefore, application of an anthocyanin-based system might disturb the endogenous anthocyanin biosynthesis. As a fluorescent protein from jellyfish (*Aequorea victoria*), GFP does not participate in plant biological processes. Recently, like *Ros1*, the *GFP* gene was also overexpressed in tomato driven by the 35S promoter, and its subsequent silencing was used as a traceable marker to indicate the occurrence of gene silencing (Quadrana *et al.*, 2011). This GFP-based system was also shown to have only a negligible impact on the metabolism in leaves and fruits (Quadrana *et al.*, 2011). In the present experiments, no obvious phenotypic changes were found in any tested plant (data not shown). Furthermore, similarly to anthocyanin, the concentration of GFP protein was also able to reflect the viral load and degree of silencing. Therefore, it is possible to perform high-throughout pre-screening by GFP-based enzymed-linked immunosorbent assay (ELISA) analysis.

In the past decade, a large numbers of genes were identified from economically important plants, such as tomato (Tomato Genome Consortium, 2012), cucumber (Huang *et al.*, 2009), apple (Velasco *et al.*, 2010), strawberry (Shulaev *et al.*, 2011), and rose (Dubois *et al.*, 2012; Kim *et al.*, 2012; Pei *et al.*, 2013). These plants all possess some special traits which cannot be investigated in model plants, such as fruit ripening, floral colour, and fragrance. Therefore, it is crucial to perform functional characterization of genes in these plants themselves instead of in model plants, such as *Arabidopsis*. This study suggested that the modified TRV–GFP vector is a simple but effective tool for functional genomics, especially in non-Solanaceae plants.

## Supplementary data

Supplementary data are available at *JXB* online.

Figure S1. Validation of the TRV–GFP vector in *N. benthamiana* by using the axil injection method (syringe with needle).

Figure S2. Validation of the TRV–GFP vector in detached rose petals.

Table S1. Primers used for vector construction and RT–PCR.

Supplementary Data
