# Supplementary material for: TRV–GFP: a modified Tobacco rattle virus vector for efficient and visualizable analysis of gene function
Source: J Exp Bot. 2013 Nov 11;65(1):311–22. doi: 10.1093/jxb/ert381 (PMC3883300; doi:10.1093/jxb/ert381)
Supplement: Supplementary Data [file supp_65_1_311__index.html]

TRV–GFP: a modified Tobacco rattle virus vector for efficient and visualizable analysis of gene function — TRV–GFP: a modified Tobacco rattle virus vector for efficient and visualizable analysis of gene function — Supplementary Data 

# TRV–GFP: a modified *Tobacco rattle virus* vector for efficient and visualizable analysis of gene function

## Supplementary Data

Data files

**Files in this Data Supplement:**

- Supplementary Data - Supplementary Data
