# Supplementary material for: TRV–GFP: a modified Tobacco rattle virus vector for efficient and visualizable analysis of gene function
Source: J Exp Bot. 2013 Nov 11;65(1):311–22. doi: 10.1093/jxb/ert381 (PMC3883300; doi:10.1093/jxb/ert381)
Supplement: Supplementary Data [file supp_ert381_jexbot105858_file001.pdf]

Supplementary Table

Supplementary Table S1. Oligo Primers used.

ID	Sequence	Purpose	Note
8892-8921 upper *Hind* III	GCCAAGCTTGCATGCCTGCAGGTCAACATG	TRV-GFP generation	
1655-1679 lower *BamH* I	ACCGGATCCCCATGGAGGCCTTCTA	TRV-GFP generation	
1161-1172 *EGFP* upper overlap	CGTCCTAATCCCATGGTGAGCAAGGGC	TRV-GFP generation	underline indicates EGFP sequence
1157-1172 *EGFP* lower overlap	TGCTCACCATGGGATTAGGACGTATC	TRV-GFP generation	underline indicates EGFP sequence
EGFP 1176-1194 upper overlap	GTACAAGTAAGGATTTAAGGACGTGAACT	TRV-GFP generation	underline indicates EGFP sequence
EGFP 1176-1187 lower overlap	GTCCTTAAATCCTTACTTGTACAGCTCGTC	TRV-GFP generation	underline indicates EGFP sequence
NtPDS upper 1	CTGACGAGCTTTCGATGCAGTGCAT	*NtPDS* silencing vector	
NtPDS lower 1	ATATATGGACATTTATCACAGGAAC	*NtPDS* silencing vector	
RhPDS upper 1	ATTTCTAGAGGACTGAACTCCGTCGTTCT	*RhPDS* silencing vector	
RhPDS lower 1	ATTGAGCTCCGAAAAGTTACCCTTGCC	*RhPDS* silencing vector	
TRV1 upper	TTACAGGTTATTTGGGCTAG	RT-PCR	
TRV1 lower	CCGGGTTCAATTCCTTATC	RT-PCR	
TRV2 upper	TGGGAGATGATACGCTGTT	RT-PCR	
TRV2 lower	CCTAAAACTTCAGACACG	RT-PCR	
*NtPDS*upper 2	TCCAAGTGCCACGACCCGAAGA	RT-PCR	

NtPDS lower 2	CAAAGCGGCTGAACTCCCCTGG	RT-PCR
NtACT upper	AGGGTTTGCTGGAGATGATG	RT-PCR
NtACT lower	CGGGTTAAGAGGTGCTTCAG	RT-PCR
RhPDS upper 2	CGTTCTGGCGAACAACGAAG	RT-PCR
RhPDS lower 2	TGAAGCATGCACCCAACTCT	RT-PCR
RhACT5 upper	AGGGTTTGCTGGAGATGATG	RT-PCR
RhACT5 lower	CGGGTTAAGAGGTGCTTCAG	RT-PCR
GFP upper	ATGGTGAGCAAGGGCGAGGA	RT-PCR
GFP lower	CTTGTACAGCTCGTCCATGCC	RT-PCR
CP-GFP fusion upper	CGATGATGCCTCTACAGCTTTCC	RT-PCR
CP-GFP fusion lower	GGCTGTTGTAGTTGTACTCCAGC	RT-PCR
AtACT2 upper	TGTTCCCAAGTATTGTTGGTCGTC	RT-PCR
AtACT2 lower	GCTGAGGGATGCAAGGATTGATC	RT-PCR
DgUbi upper	CTAATGAATGCTTACTGTGACCGAC	RT-PCR
DgUbi lower	AGGCGAATCATCAGTACCAAGTG	RT-PCR
FaACT upper	TGGGTTTGCTGGAGATGAT	RT-PCR
FaACT lower	CAGTAGGAGAACTGGGTGC	RT-PCR

Supplementary Figures

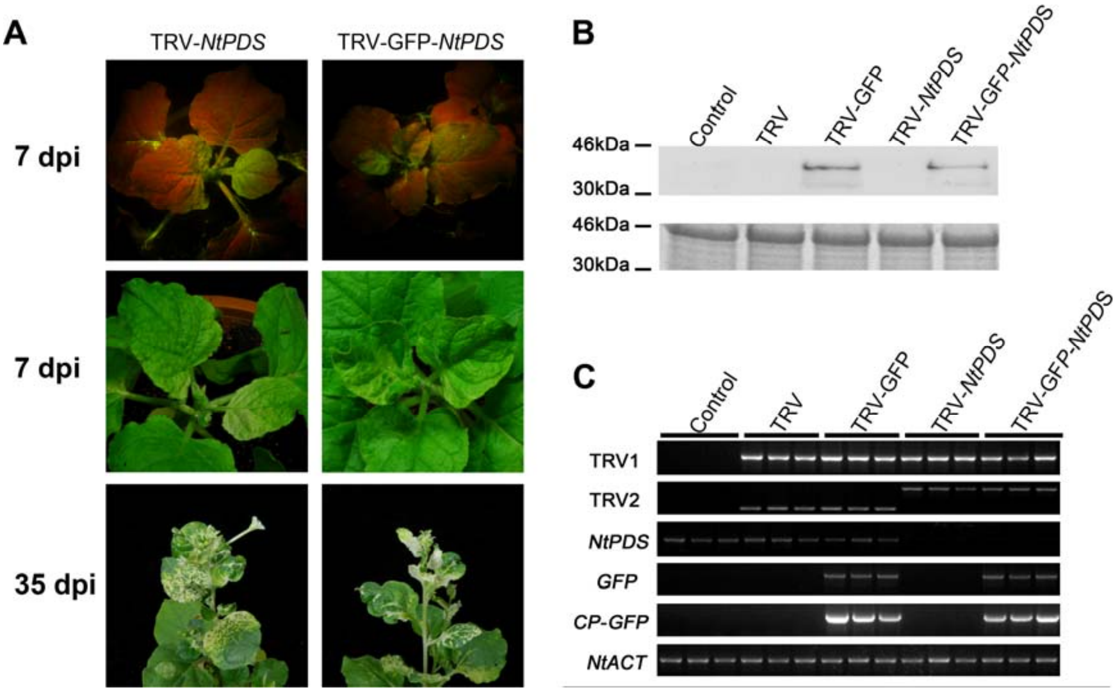

Figure S1. Validation of TRV-GFP vector in *N. benthamiana*. (A) TRV and TRV-GFP infiltrated plants. The 4-leaves stage plants were inoculated on axil by mixture of pTRV2-*NtPDS*/ pTRV2-GFP-*NtPDS* and pTRV1 (1:1, OD600=4.0) using a syringe with needle. Plants were photographed under UV illumination and normal light. (B) CP-GFP protein levels in upper leaves 7 days after TRV vector inoculation. Ten microgram protein was used for Western blot in each lane and anti-GFP was used as antibody to detect CP-GFP fusion protein. Coomassie blue staining was used for confirmation of equal loading in each lane. (C) Semi-quantitative RT-PCR of *TRV1, TRV2*, *NtPDS*, *GFP* and *CP-GFP* in control and inoculated plants. *NtACT* was used as internal control.

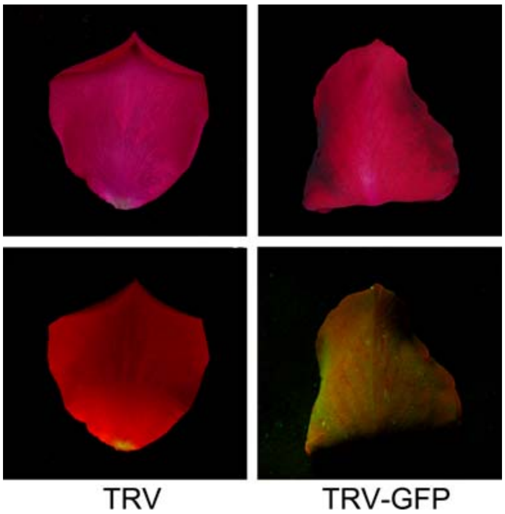

Figure S2. Validation of TRV-GFP in detached rose petals. Rose petals were inoculated by mixture of pTRV1 and pTRV2 (left) or pTRV1-GFP (right) (1:1, OD600=1.0) using vaccum-infiltration. The petals were photographed under normal light (upper panel) and UV illumination (bottom panel) at 7 dpi, respectively.
